# Membrane Potential Determined by Flow Cytometry Predicts Fertilizing Ability of Human Sperm

**DOI:** 10.3389/fcell.2019.00387

**Published:** 2020-01-21

**Authors:** Lis C. Puga Molina, Stephanie Gunderson, Joan Riley, Pascal Lybaert, Aluet Borrego-Alvarez, Emily S. Jungheim, Celia M. Santi

**Affiliations:** ^1^Department of Obstetrics & Gynecology, Washington University School of Medicine, St Louis, MO, United States; ^2^Laboratory of Experimental Hormonology, School of Medicine, Université Libre de Bruxelles, Brussels, Belgium

**Keywords:** membrane potential, human, sperm, IVF, normozoospermic infertility, flow cytometry, capacitation

## Abstract

Infertility affects 10 to 15% of couples worldwide, with a male factor contributing up to 50% of these cases. The primary tool for diagnosing male infertility is traditional semen analysis, which reveals sperm concentration, morphology, and motility. However, 25% of infertile men are diagnosed as normozoospermic, meaning that, in many cases, normal-appearing sperm fail to fertilize an egg. Thus, new information regarding the mechanisms by which sperm acquire fertilizing ability is needed to develop a clinically feasible test that can predict sperm function failure. An important feature of sperm fertilization capability in many species is plasma membrane hyperpolarization (membrane potential becoming more negative inside) in response to signals from the egg or female genital tract. In mice, this hyperpolarization is necessary for sperm to undergo the changes in motility (hyperactivation) and acrosomal exocytosis required to fertilize an egg. Human sperm also hyperpolarize during capacitation, but the physiological relevance of this event has not been determined. Here, we used flow cytometry combined with a voltage-sensitive fluorescent probe to measure absolute values of human sperm membrane potential. We found that hyperpolarization of human sperm plasma membrane correlated positively with fertilizing ability. Hyperpolarized human sperm had higher *in vitro* fertilization (IVF) ratios and higher percentages of acrosomal exocytosis and hyperactivated motility than depolarized sperm. We propose that measurements of human sperm membrane potential could be used to diagnose men with idiopathic infertility and predict IVF success in normozoospermic infertile patients. Patients with depolarized values could be guided toward intracytoplasmic sperm injection, preventing unnecessary cycles of intrauterine insemination or IVF. Conversely, patients with hyperpolarized values of sperm membrane potential could undergo only conventional IVF, avoiding the risks and costs associated with intracytoplasmic sperm injection.

## Introduction

Infertility, defined as the inability of a couple to achieve pregnancy after 12 months of unprotected intercourse, affects between 10 and 15% of couples around the world (Sharma et al., 2013). Many such couples turn to *in vitro* fertilization (IVF), which has been used to conceive over 6.5 million babies. Approximately 50% of infertility cases are due to a male factor (Kumar and Singh, 2015). To diagnose these men, assisted reproduction specialists rely on semen analysis, which provides information about sperm concentration, morphology, and motility. However, this method does not reveal abnormalities in sperm from some infertile men (Bracke et al., 2018), who are then described as having normozoospermic idiopathic infertility. One possibility is that sperm from these men are unable to fertilize an egg because they are unable to capacitate, a process in which sperm become hyperactive and prepared to undergo acrosomal exocytosis. Together, hyperactivation and acrosomal exocytosis allow sperm to bind to and fuse with an oocyte (Yanagimachi, 1994). In natural pregnancies, capacitation is triggered by factors in the female reproductive tract (Austin, 1951; Chang, 1951). In IVF, sperm is capacitated by incubation in a chemically defined media containing Ca^++^, HCO_3_^–^, energy sources, and a cholesterol acceptor. Given that capacitation is required for fertilization, a test that could assess the ability of sperm to undergo this process could be a valuable addition to IVF diagnostics.

In many species, sperm capacitation is accompanied by sperm plasma membrane hyperpolarization (an increase in intracellular net negative charge) (Zeng et al., 1995; Hernández-González et al., 2007; López-González et al., 2014; Escoffier et al., 2015). In mouse sperm, capacitation-associated hyperpolarization is largely driven by activation of the sperm-specific potassium (K^+^) channel SLO3 (Santi et al., 2010; Chávez et al., 2013). *Slo3* knockout mice are infertile, and their sperm are unable to undergo hyperactivation or acrosomal exocytosis (Santi et al., 2010; Zeng et al., 2011). Human sperm also hyperpolarize during capacitation (López-González et al., 2014), and two studies reported that more depolarized human sperm membrane potentials values were associated with lower fertility (Calzada and Tellez, 1997; Brown et al., 2016). However, these studies did not address the effect of changes of sperm membrane potential on hyperactivation or acrosomal exocytosis. In addition, the methods used in both studies to assess membrane potential are technically difficult and not suitable to be implemented in a clinical setting. Other methods that evaluate the capacitating state of the sperm, such as the CapScore test, require staining, counting, and correctly identifying staining patterns in more than 150 individual sperm (Moody et al., 2017), which also is challenging to implement clinically.

Here, we used flow cytometry in combination with the voltage-sensitive fluorescent dye DiSC_3_(5) to create a calibration curve and measure absolute sperm membrane potential values. Using this method, we found that human sperm membrane hyperpolarization correlated with sperm fertility capacity in IVF patients. We propose that our method could be used clinically to diagnose male infertility and predict IVF success of normozoospermic patients.

## Materials and Methods

### Reagents

All reagents to prepare non-commercial Human Tubal Fluid (HTF) and Toyoda–Yokoyama–Hosi (TYH) media, as well as valinomycin, FITC-labeled *Pisum sativum* agglutinin, Tween 20, 2′,7′-Bis-(2-Carboxyethyl)-5-(and-6)-Carboxyfluorescein, Acetoxymethyl Ester (BCECF-AM), and calcium ionophore A23187, were from Sigma (St. Louis, MO); 3, 3′-dipropyl- thiadicarbocyanine iodide [DiSC_3_(5)] was from Invitrogen (Carlsbad, CA, United States); Hoechst 33342 was from Cayman Chemicals, (Ann Arbor, MI, United States); paraformaldehyde was from Electron Microscopy Sciences (Hatfield, PA, United States); Dulbecco’s Phosphate Buffered Saline (DPBS) was from GIBCO Life Technologies (Gaithersburg, MD, United States); and Quinn’s Advantage Human (HTF) fertilization media and human serum albumin were from CooperSurgical (Trumbull, CT, United States).

### Mouse Samples and Ethics Statement

Washington University School of Medicine (WUSM) policy states that all research involving animals be conducted under humane conditions, with appropriate regard for animal welfare. WUSM is a registered research facility with the United States Department of Agriculture (USDA) and is committed to comply with the Guide for the Care and Use of Laboratory Animals (Department of Health and Human Services) and the provisions of the Animal Welfare Act (USDA and all applicable federal and state laws and regulations). The WUSM Animal Care and Use Committee ensures compliance with all applicable federal and state regulations for the purchase, transportation, housing, and research use of animals. WUSM has filed appropriate assurance of compliance with the Office for the Protection of Research Risks of the National Institute of Health. The WUS Animal Care and Use Committee approved the method of euthanasia for mice: CO_2_ asphyxiation followed by rapid cervical dislocation. These methods are consistent with the recommendation of the panel on euthanasia of the American Veterinary Medical Association and YARC Standard Euthanasia guidelines for rodents. Caudal epididymal sperm were collected from 90-day-old *Slo3* knock-out (generated as previously described [Santi et al., 2010]) or wild-type mice.

### Human Samples and Ethics Statement

This study was approved by the Washington University Institutional Review Board and was performed in collaboration with the Washington University Fertility and Reproductive Medicine Center. Semen samples were obtained via masturbation after 3–5 days of abstinence in private collection rooms at the fertility clinic. Only donors and conventional IVF patients’ samples that met the World Health Organization [WHO] (2010) (≥32% progressive motility; ≥40% total motility; ≥15 × 10^6^ cells/ml). For studies involving semen donors, de-identified human sperm samples were obtained within 2 h of collection from men who were asked to have a semen analysis performed.

Couples undergoing IVF met inclusion criteria if their oocytes were subjected to conventional *in vitro* fertilization or split insemination [half intracytoplasmic sperm injection (ICSI), half conventional *in vitro* fertilization] or conventional *in vitro* fertilization, and if the female partner had a diagnosis of unexplained infertility, tubal infertility, or uterine factor infertility. Patients were excluded from this study if they had male factor infertility. Additionally, to assure good-quality oocytes for this study, patients were excluded if females had polycystic ovarian syndrome, endometriosis, anovulation, or diminished ovarian reserve. The average age of the 49 healthy men included in this study was 34.63 (range 26–48) years. On the day of oocyte retrieval, male partners gave written informed consent in accordance with the Declaration of Helsinki. At the time of consent, male partners also completed a brief health questionnaire. For IVF, around 14 × 10^6^/ml sperm were incubated overnight with mature oocytes including the zona pellucida and cumulus cells. For ICSI, oocytes with zona pellucida and without cumulus cells were used. Sperm samples left over after IVF were obtained approximately 4 h after initial collection and incubated overnight at 37°C with 5% CO_2_. IVF cycle data including fertilization rate were collected for analysis. The fertilization rate was calculated as the number of normally fertilized mature oocytes with two pronuclei divided by total number of inseminated mature oocytes. The Washington University Fertility and Reproductive Medicine Center defines successful IVF as a fertilization rate >70% (fertilization ratio >0.7); this cut-off is determined by calculating the mean fertilization ratio in conventional IVF cases quarterly and yearly.

For studies involving frozen human samples, five vitrified washed normozoospermic semen samples that were used to conceive a pregnancy were obtained from the Washington University Reproductive Medicine Center cryobank. The samples were produced by anonymous donors, aliquoted, frozen, and used for both intrauterine insemination and IVF. Remaining aliquots of samples that were used to conceive a pregnancy were donated for the purposes of research.

### Media

Non-commercial HEPES-buffered HTF (4.67 mM KCl, 0.31 mM KH_2_PO_2_, 90.69 mM NaCl, 1.20 mM MgSO_4_, 2.78 mM glucose, 1.6 mM CaCl_2_, 0.51 mM sodium pyruvate, 60 mM sodium lactate, 23.8 mM HEPES, and 10 μg/ml Gentamicin, (pH 7.4 with NaOH) was used to measure human sperm membrane potential. Non-commercial HEPES-buffered HTF was supplemented the same day with 25 mM NaHCO_3_ and 0.5% w/v BSA A-7906 (Sigma) for capacitating conditions, and only with 0.5% w/v BSA for non-capacitating conditions. IVF samples were processed in commercial Quinn’s Advantage Human (HTF) fertilization media [CooperSurgical (Trumbull, CT, United States)] supplemented with 3 mg/ml human serum albumin (CooperSurgical) and pre-incubated overnight at 37°C with 5% CO_2_ to equilibrate pH. The same media was used to capacitate IVF and donor sperm samples.

Mouse capacitating modified (TYH) medium [119.30 mM NaCl, 4.70 mM KCl, 1.71 mM CaCl_2_, 1.20 mM KH_2_PO4, 1.20 mM MgSO_4_, 0.51 mM sodium pyruvate, 5.56 mM glucose, 20 mM HEPES, 15 mM NaHCO_3_ and 5 mg/ml BSA A-2153 (pH 7.4 with NaOH)] was used to capacitate mouse sperm.

### Human and Mouse Sperm Capacitation

Ejaculated human sperm were allowed to swim-up in commercial or non-commercial HTF capacitating medium for 1 h at 37°C with CO_2_. The highly motile sperm recovered after swim up were washed, centrifuged for 5 min at 400 × *g*, and incubated overnight at 37°C with 5% CO_2_. For experiments involving comparisons between non-capacitating and capacitating conditions, human sperm were allowed to swim-up in non-capacitating HTF medium at 37°C for 1 h. Then the sample was divided into two, and an equal volume of non-capacitating medium or 2-fold concentrated BSA and NaHCO_3_ were added to prepare non-capacitating or capacitating media, respectively. Then, the samples were incubated overnight at 37°C with 5% CO_2_ for capacitating conditions, or at 37°C for non-capacitating conditions.

Frozen human sperm samples were thawed for 20 min at room temperature, resuspended in 1.5 ml final volume of commercial Quinn’s Advantage Human (HTF) fertilization media (CooperSurgical) medium and washed twice with centrifugation at 400 × *g* for 5 min. Sperm were allowed to swim-up in 300 μl of media with 5% CO_2_ at 37°C for 1 h. Highly motile sperm were recovered from the supernatant. Half were used to determine membrane potential, and half were incubated overnight with 5% CO_2_ at 37°C for to determine membrane potential after capacitation.

Mice were euthanized, and cauda epididymal sperm were placed in 1 ml of modified TYH medium. After 15 min at 37°C (swim-out), suspended sperm were collected. Sperm concentration was adjusted to a final maximum concentration of 10 × 10^6^ cells/ml and incubated for 75 min at 37°C for capacitation.

### Determination of Membrane Potential by Flow Cytometry

After overnight incubation, human sperm samples were centrifuged at 400 × *g* for 5 min and resuspended in non-capacitating HTF medium and 0.1 μM BCECF-AM. The samples were covered to be protected from light, incubated at 37°C for 10 min, washed again to eliminate non-incorporated dye, and resuspended in non-capacitating HTF media with 25 mM NaHCO_3_. Before recording, 5 nM DiSC_3_(5) was added, and data were recorded as individual cellular events on a FACSCanto II TM cytometer (Becton Dickinson). Forward scatter area (FSC-A) and side-scatter area (SSC-A) data were collected from 20,000 events per recording. Threshold levels of two-dimensional dot plot SSC-A vs. side-scatter height (SSC-H) were set for gating singlets. BCECF fluorescence was detected with the filter for fluorescein isothiocyanate (FITC; 530/30) and used to exclude non-viable cells as previously described (Puga Molina et al., 2017). Positive cells for DiSC_3_(5) fluorescence was detected with the filter for allophycocyanin (APC; 585/40). Because DiSC_3_(5) is positively charged, cell hyperpolarization increases dye incorporation and intracellular fluorescence. Recordings were initiated after reaching steady-state fluorescence (1–3 min).

Mouse sperm samples were centrifuged at 200 × g for 10 min and resuspended in TYH media without BSA. Before recording, 5 nM DiSC_3_(5) and 900 nM Hoechst 33342 were added. Data were recorded as individual cellular events on a FACSCanto II TM cytometer (Becton Dickinson). FSC-A and SSC-A data were collected from 20,000 events per recording. Threshold levels of two-dimensional dot plot SSC-A vs. SSC-H were set for gating singlets. Hoechst 33342 fluorescence was detected with the filter for pacific blue (Pacific Blue; 450/50) and used to exclude non-viable cells. DiSC_3_(5) fluorescence was detected with the filter for allophycocyanine (APC; 585/40).

In both mouse and human sperm experiments, membrane potential fluorescent signal was calibrated by adding 1 μM valinomycin and sequential adding KCl corresponding to 4.98, 7.48, 12.48, 22.48, and 42.48 mM K^+^ for HTF and 5.90, 8.4, 13.4, 23.4, and 43.4 mM K^+^ for TYH (Chávez et al., 2013). Theoretical values were obtained by using the Nernst equation, assuming an intracellular K^+^ concentration of 120 mM (Linares-Hernández et al., 1998) and considering room temperature as 298.15°K. The final sperm membrane potential was obtained by linearly interpolating the median fluorescence of the unknown sample to the calibration curve of each trace.

### Determination of Membrane Potential by Spectrophotometry

After swim-up incubation, human sperm samples were centrifuged at 400 × *g* for 5 min, resuspended in non-capacitating HTF media with 5 mg/ml of BSA A-2153 (Sigma), and 4 × 10^6^ sperm were transferred to a gently stirred cuvette at 310.15^°^K. DiSC_3_(5) was added to a final concentration of 1 μM. Fluorescence was monitored with a Varian Cary Eclipse fluorescence spectrophotometer at 620 nm excitation and 670 nm emission as described before (Chávez et al., 2013). Calibration was performed by adding 1 μM valinomycin and sequential additions of KCl as described for flow cytometry experiments. The final sperm membrane potential was obtained by linearly interpolating the theoretical membrane potential values versus arbitrary units of fluorescence of each trace.

### Acrosomal Status

To assess acrosomal status, 50 μl human sperm samples were treated with 10 μM of the calcium ionophore A23187 or DMSO (vehicle) for 30 min before the end of the capacitation incubation time. After incubation, sperm were fixed at 4°C by adding 50 μl of 12.5% w/v paraformaldehyde in 2.28 M Tris. Sperm were then spotted onto slides, dried, washed 3 times for 5 min and one time for 15 min with DPBS containing 0.1% v/v Tween 20 (t-DPBS), and stained with 100 μg/ml of FITC-labeled *Pisum sativum* agglutinin dissolved in t-DPBS. After washing, slides were observed under an EVOS^®^ FL Cell Imaging System epifluorescence microscope at 40×. Sperm were scored as acrosome intact if a bright staining was observed in the acrosome, or as acrosome reacted when either fluorescent staining was restricted to the equatorial segment or no labeling was observed. The percentage of sperm that underwent induced acrosomal exocytosis was obtained by subtracting the percentage of acrosome reacted sperm incubated with DMSO from the percentage of acrosome reacted sperm incubated with A23187.

### Computer-Assisted Sperm Analysis (CASA)

Aliquots (3 μl) of sperm suspension were placed into a 20 micron Leja standard count 4 chamber slide, pre-warmed at 37°C. CASA analysis was performed with a Hamilton–Thorne digital image analyzer (HTR-CEROS II v.1.7; Hamilton–Thorne Research, Beverly, MA, United States). Phase alignment was checked, and the settings used for the analysis were selected as follows: objective 1: Zeiss 10XNH; min total count: 200; frames acquired, 30; frame rate, 60 Hz; camera exposure: 8 ms; camera gain: 300; integrated time: 500 ms; elongation max%: 100; elongation min%: 1; head brightness min 170; head size max: 50 μm^2^; head size min: 5 μm^2^; static tail filter: false; tail brightness min: 70; tail brightness auto offset: 8; tail brightness mode: manual; progressive STR (%): 80; progressive VAP (μm/s): 25. The criteria used to define hyperactivated sperm were: curvilinear velocity (VCL) >150 μm/s, lateral head displacement (ALH) >7.0 μm, and linearity coefficient (LIN) <50% (Mortimer et al., 1998).

### Calculations and Statistical Analyses

Data are expressed as mean ± standard error of the mean (SEM). Independent experiments were carried out with samples from different donors or patients. A probability (*p*) value *p* < 0.05 was considered statistically significant. A value of *p* < 0.05 was indicated with an ^∗^, *p* < 0.01 with ^∗∗^, and *p* < 0.001 with ^∗∗∗^. Calculations were performed with Microsoft Office 365 ProPlus spreadsheet, and statistical analyses were performed with GraphPad Prism version 6.01, GraphPad Software (La Jolla, CA, United States). To test whether values were in a Gaussian distribution, D’Agostino-Pearson, Shapiro-Wilk, and Kolmogorov-Smirnov tests were run in parallel. Parametric or non-parametric comparisons were used as dictated by the data distribution. Spearman test was used for correlations. The differences between means from two groups were analyzed by paired or unpaired *t*-test, depending on the condition tested. The receiving operating characteristic (ROC) curve and the maximum Youden index were used to determine the optimal cut-off value for depolarized and hyperpolarized samples.

## Results

### Flow Cytometry Can Be Used to Measure Absolute Values of Sperm Membrane Potential

**FIGURE 1:**
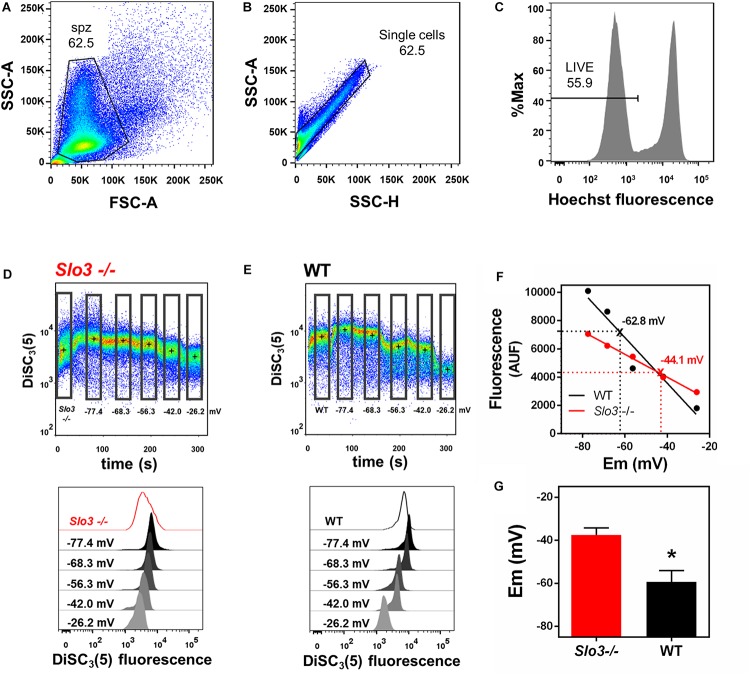
Flow cytometry can be used to determine absolute values of membrane potential in wild type and *Slo3*^–/–^ mouse sperm. **(A)** Forward-scatter (FSC) and side-scatter (SSC) light data were collected. Threshold levels for side-scatter area (SSC-A) and forward-scatter area (FSC-A) were set to exclude signals from cellular debris or cells with abnormal morphology. **(B)** Threshold levels of two-dimensional dot plot of SSC-A vs. side-scatter height (SSC-H) were set to include only single cells and exclude clumps. **(C)** Cells with bright Hoechst staining were excluded to measure DiSC_3_(5) fluorescence only in live sperm. **(D,E)** Upper panel: Representative dot plot of DiSC_3_(5) fluorescence from *Slo3*^–/–^ (**D**) and WT (**E**) mouse sperm incubated in capacitated conditions. Calibration was performed by adding 1 μM valinomycin and sequentially adding KCl, corresponding to the theoretical plasma membrane potential values of –77.4, –68.3, –56.3, –42.0, and –26.2 mV ([K^+^]_i_ of 120 mM). Black crosses indicate the median DiSC_3_(5) fluorescence of sperm within the gates (rectangles) defined for each addition. Lower panels: Histograms of DiSC_3_(5) fluorescence from selected gates as indicated in flow cytometry dot plots. **(F)** Linear regression of the calibration curves constructed from the median DiSC_3_(5) fluorescence from *Slo3*^–/–^ and WT sperm obtained in D and E. Interpolated values for each curve are indicated with X and dotted lines. *Slo3*^–/–^ *r*^2^ = 0.9948; WT *r*^2^ = 0.9311. **(G)** Membrane potential of *Slo3*^–/–^ and WT capacitated sperm. ^∗^*P* = 0.0265 by unpaired *t*-test.

Our method of calculating absolute sperm membrane potential values is based on the properties of the cationic voltage-sensitive dye DiSC_3_(5), which distributes across cellular membranes in response to electrochemical gradients. DiSC_3_(5) enters the sperm upon membrane hyperpolarization, increasing intracellular fluorescence. DiSC_3_(5) has been used to measure mammalian sperm membrane potential by spectrophotometry (Espinosa and Darszon, 1995; Muñoz-Garay et al., 2001; Demarco et al., 2003; Santi et al., 2010). Since flow-cytometry in association with DiSC_3_-(5) was not previously used to determine absolute sperm membrane potential values, we first validated our method. To do so, we conducted DiSC_3_(5) flow cytometry experiments with both wild type (WT) and *Slo3*^–/–^ mouse sperm and compared our values to those previously measured by spectrophotometry (Santi et al., 2010). Forward scatter area (FSC-A) and side scatter area (SSC-A) light detection were used to identify sperm cells based on size and granularity (Figure 1A). To exclude non-single cells (doublets or debris), singlets were selected with SSC-A and side-scatter height (SSC-H) (Figure 1B; Escoffier et al., 2015). Hoechst 33342 dye was used to differentiate between live and dead sperm (Figure 1C), and DiSC_3_(5) fluorescence was analyzed only in live sperm (see section Materials and Methods).

To calibrate the fluorescent signal, we constructed a calibration curve by using the K^+^ ionophore valinomycin and sequential additions of known concentrations of KCl. Addition of valinomycin causes membrane hyperpolarization up to the value of the K^+^ equilibrium potential, causing DiSC_3_(5) to enter the sperm, leading to increased intracellular fluorescence. Sequential KCl additions cause depolarization of the plasma membrane to the corresponding K^+^ equilibrium potential values and decreased intracellular fluorescence. The membrane potential values after addition of valinomycin and KCl were calculated according to the Nernst equation, assuming a [K^+^]_i_ of 120 mM (Figures 1D,E). The absolute sperm membrane potential values were obtained by linearly interpolating the theoretical membrane potential values versus arbitrary units of fluorescence of each trace (Figure 1F). The membrane potential values obtained with this technique from WT (−59.4 ± 5.3 mV) and *Slo3*^–/–^ (−37.5 ± 3.3 mV) sperm (Figure 1G) were similar to those reported by spectrophotometry by our group (−60 mV for WT and −40 mV for *Slo3*^–/–^) (Santi et al., 2010). Thus, we concluded that our flow cytometric method of measuring absolute membrane potential values was valid for mouse sperm.

### Human Sperm Incubated in Capacitation Conditions Are More Hyperpolarized Than Non-capacitated Sperm

**FIGURE 2:**
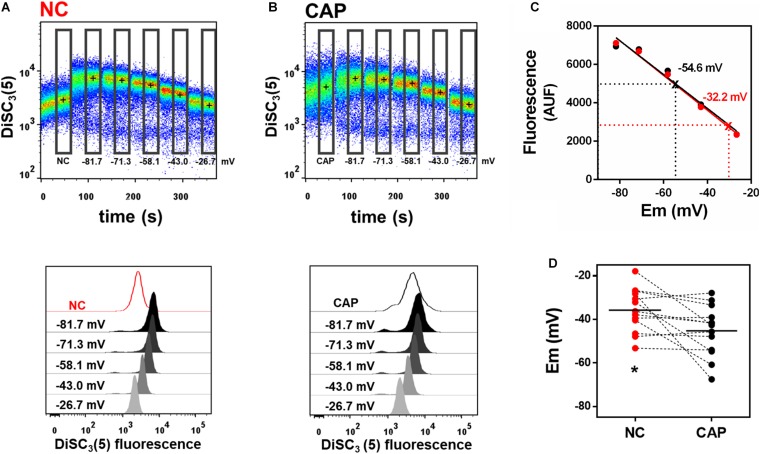
Incubation in capacitating media hyperpolarizes human sperm. **(A,B)** Upper panel: Representative dot plot of DiSC_3_(5) fluorescence traces from human donor sperm incubated in non-capacitated (**A**, “NC”) or capacitating (**B**, “CAP”) HTF media. Black crosses indicate the median DiSC_3_(5) fluorescence of sperm within the gates (rectangles) defined for each addition. Calibration was performed as in Figure 1 with 1 μM valinomycin and sequential additions of KCl, corresponding to the theoretical plasma membrane potential values of –81.7, –71.3, –58.1, –43.0, and –26.7 mV. Lower panels: Histograms of DiSC_3_(5) fluorescence from selected gates as indicated in flow cytometry dot plots above. **(C)** Linear regression of the calibration curves built from the median fluorescence from NC and CAP sperm obtained in panels **A,B**. Interpolated values for each curve are indicated. NC *r*^2^ = 0.9670, CAP *r*^2^ = 0.9860. **(D)** Sperm membrane potential values in both NC and CAP media were obtained by linearly interpolating the theoretical membrane potential values versus arbitrary units of fluorescence of each trace. Dashed lines connect the same samples incubated in NC and CAP media. ^∗^*P* = 0.0212 by paired *t*-test.

Previous flow cytometry studies of human sperm membrane potential showed that non-capacitated sperm were more depolarized than sperm incubated in capacitating media (López-González et al., 2014; Brukman et al., 2019), but absolute membrane potential values were not reported. Here, we used our flow cytometric technique to measure the absolute membrane potential values in both capacitated and non-capacitated sperm from normozoospermic donors. The fluorescence probe BCECF-AM was used as an indicator of live sperm, and only BCECF-positive cells were analyzed (Puga Molina et al., 2017, 2018b; Supplementary Figures S1A–C). We conducted a calibration curve as we had done for mouse sperm (Figures 2A–C). We found that human sperm incubated in capacitation conditions were, on average, significantly more hyperpolarized than non-capacitated sperm (−45.2 ± 3.2 vs. −35.7 ± 2.8 mV, *n* = 13, respectively) (Figure 2D). However, some donor samples did not undergo hyperpolarization in capacitating media.

The spectrophotometry assay is widely used to measure absolute membrane potential values in sperm (Linares-Hernández et al., 1998; Patrat et al., 2002; Santi et al., 2010; Chávez et al., 2013). Therefore, we compared the membrane potential value obtained by spectrophotometry and flow cytometry in a donor sample. We obtained similar membrane potential values with the two techniques (Supplementary Figure S1). This result further indicates that our flow cytometric method could be used to determine human sperm membrane potential.

### Human Sperm Membrane Potential Is a Good Indicator of the Ability of Sperm to Undergo Sapacitation

**FIGURE 3:**
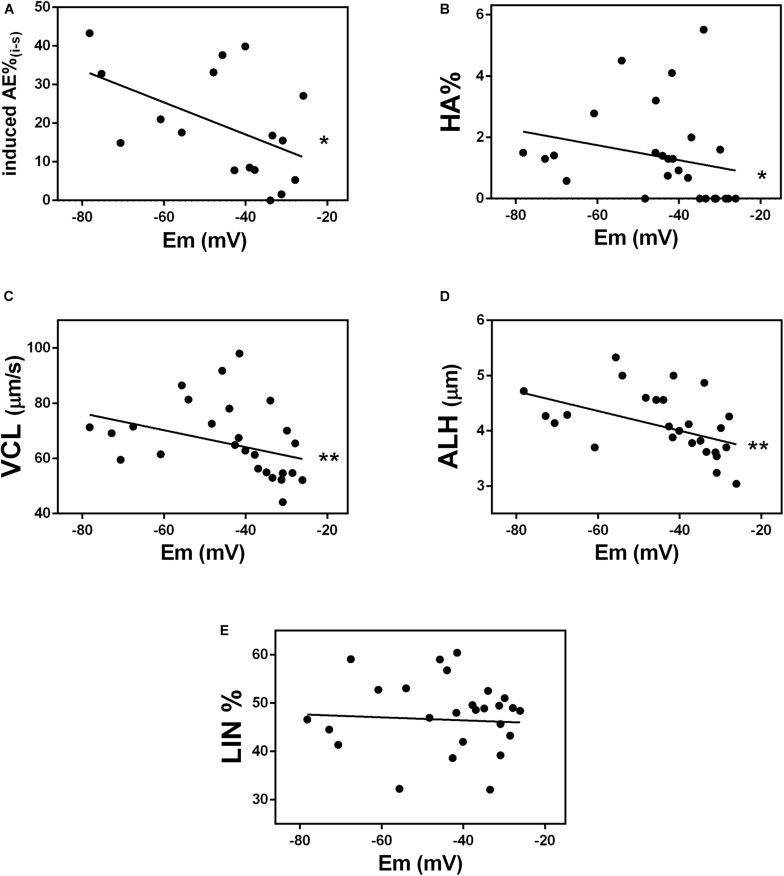
Membrane potential correlates with events related to capacitation. Correlation between absolute values of membrane potential (Em) of donor sperm incubated in HTF non-commercial capacitating media and the following: **(A)** acrosomal exocytosis (AE) induced by 10 μM A23187 (^∗^*P* = 0.0366); **(B)** hyperactivated motility (HA%) (*r* = –0.4865, ^∗^*P* = 0.0101); **(C)** curvilinear velocity (VCL) (*r* = –0.5000, ^∗∗^*P* = 0.0079); **(D)** lateral head displacement (ALH) (*r* = –0.5497, ^∗∗^*P* = 0.0030), and **(E)** linearity coefficient (LIN) (*r* = –0.0464, *P* = 0.8182).

Mouse sperm that do not hyperpolarize, do not hyperactivate and do not undergo regulated acrosomal exocytosis, the two endpoints of sperm capacitation (Demott and Suarez, 1992; Yanagimachi, 1994; Buffone et al., 2009). To determine whether capacitated human sperm membrane potential correlated with the ability of sperm to undergo acrosomal exocytosis, we used our flow cytometry method to calculate membrane potential in 17 capacitated donor normozoospermic sperm samples. In parallel, we treated the sperm with the calcium ionophore A23187, which induces acrosomal exocytosis (Köhn et al., 1997; Moody et al., 2017; Puga Molina et al., 2017) and determined the percentage of sperm in each sample that underwent acrosomal exocytosis. A scatter plot comparing the two values revealed that the membrane potential values obtained by flow cytometry correlated significantly with the percentage of cells that underwent acrosomal exocytosis (Figure 3A).

To determine whether capacitated sperm membrane potential correlated with sperm hyperactivation (HA), we used computer-assisted sperm analysis to measure three parameters associated with HA: curvilinear velocity (VCL), amplitude of lateral head displacement (AHL), and linearity (LIN). Sperm with values of VCL>150 μm/s, AHL>7.0 μm and LIN <50% were considered hyperactivated (Mortimer et al., 1998). The percentage of sperm in each of 27 normozoospermic donor samples, correlated significantly with membrane potential values (Figure 3B). Specifically, VCL and ALH values (Figures 3C,D), but not LIN values (Figure 3E), correlated with sperm membrane potential. Together, these data indicate that human sperm membrane potential is a good indicator of the ability of sperm to undergo capacitation.

### Membrane Potential of Capacitated Human Sperm Correlates With Fertilization Ratio in IVF Patients

**FIGURE 4:**
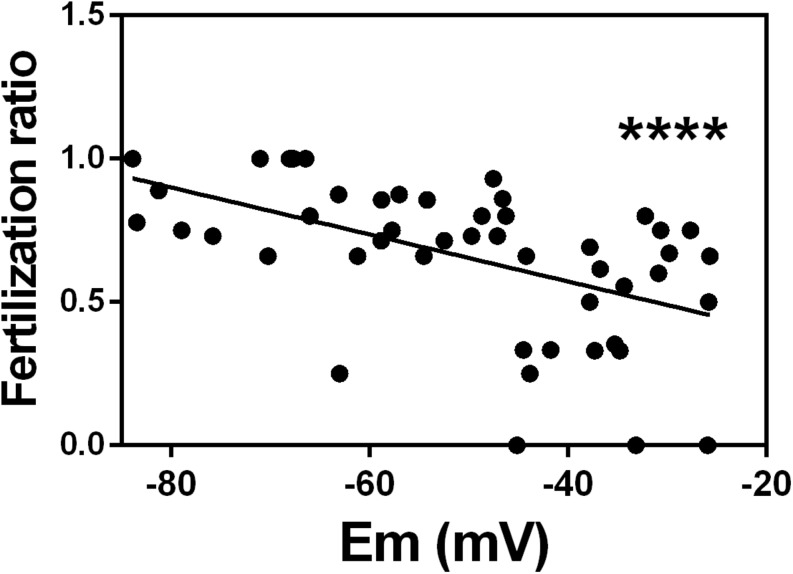
*In vitro* fertilization ratio correlates with sperm membrane potential in patients with normozoospermic infertility. Spearman correlation between sperm membrane potential (Em) from normozoospermic patients undergoing conventional *in vitro* fertilization and fertilization ratio. Fertilization ratio was calculated as the number of normally fertilized mature oocytes with two pronuclei divided by total number of inseminated mature oocytes. *n* = 49, *r* = –0.5855, ^∗∗∗∗^*P* < 0.0001.

Next, we wanted to determine whether the membrane potential could indicate the ability of sperm to fertilize an egg. To address this question, we performed our flow cytometric assay on excess samples from 49 normozoospermic men whose sperm was used for conventional IVF. We found that the percentage of fertilized oocytes (reported as fertilization ratio) from these patients significantly correlated with the absolute value of membrane potential of capacitated sperm. Those with more negative membrane potential had higher fertilization ratios than those with more positive membrane potential (Figure 4).

If IVF failure were caused by impaired sperm capacitation, this defect would be bypassed by ICSI. Thus, we reviewed the ICSI outcome of 14 patients who underwent this procedure and whose sperm resulted in an IVF ratio of less than 0.7. In three cases, the fertilization ratio was the same in ICSI and conventional IVF. However, for the other 11, the ICSI fertilization ratio was higher than the IVF ratio (mean values *n* = 14 0.8648 vs. 0.4208; Wilcoxon test, *P* = 0.0005) (Supplementary Figure 2A). This result suggests that the low IVF ratios were related to impaired sperm capacitation.

### Fertile Sperm Are Hyperpolarized

**FIGURE 5:**
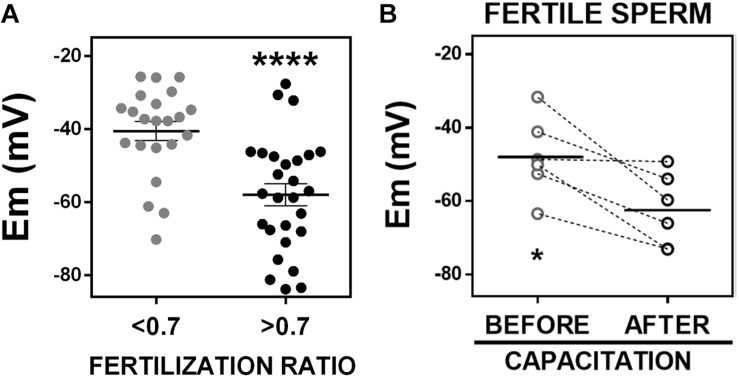
Fertile sperm are hyperpolarized. **(A)** Sperm membrane potential (Em) values from patients with *in vitro* fertilization ratios <0.7 (*n* = 22; –40.61 ± 2.60 mV) or >0.7 (*n* = 27, –58.03 ± 3.00 mV). ^∗∗∗∗^*P* < 0.0001 by unpaired *t*-test. **(B)** Membrane potential of fertile frozen sperm samples from patients that conceived a pregnancy, incubated in commercial capacitating media for 1 h (before capacitation, –47.92 ± 4.38 mV) or 18 h (after capacitation, –62.51 ± 4.05 mV). *n* = 6, ^∗^*P* = 0.0146 by paired *t*-test.

Given the correlation we observed between sperm membrane potential values and fertilization ratios in IVF, we wondered whether we could detect a significant difference in sperm membrane potential values between patients with successful and non-successful IVF, which are defined as fertilization ratio >0.7 or <0.7, respectively (Harris et al., 2019). This cut-off was determined by calculating the mean fertilization ratio in conventional IVF cases quarterly and yearly. Thus, we dichotomized the samples into those with fertilization ratio >0.7 (*n* = 27) and those with fertilization ratio <0.7 (*n* = 22) and compared their sperm membrane potential values. Whereas sperm samples with fertilization ratio >0.7 had an average membrane potential of −58.03 ± 3.00 mV, sperm samples with fertilization ratio <0.7 had an average membrane potential of −40.61 ± 2.60 mV (Figure 5A).

We next investigated whether sperm samples capable of fertilizing an egg, hyperpolarize during capacitation. These experiments require measuring the membrane potential before (time = 0 h) and after capacitation (time = 18 h). This kind of experiment was not possible to do using fresh sperm because these samples were obtained after finishing clinical procedures and were kept in capacitating media for 2–4 h. Therefore, we did not have a real 0 time point. For this reason, we decided to use and aliquot of frozen samples from anonymous donors that had resulted in pregnancies. We used flow cytometry in these frozen samples to measure membrane potential before (*t* = 0) and after capacitation (*t* = 18 h). We found that all these fertile sperm samples underwent hyperpolarization after capacitation, changing from −47.9 ± 4.4 to −62.5 ± 4.1 mV (Figure 5B). Although there could be many differences between frozen and fresh sperm samples, it is noteworthy that the mean membrane potential value of frozen sperm from fertile donors after capacitation was similar to the membrane potential value of fresh sperm samples from patients with successful IVF (fertilization ratio >0.7) (Figure 5A). In addition, the mean membrane potential values of the frozen sperm from fertile donors before capacitation, was similar to that of fresh sperm from patients with fertilization ratio <0.7. These findings suggest that infertile sperm fail to hyperpolarize in capacitating conditions: even though the samples with lower fertilization ratio were incubated in capacitated conditions, they apparently failed to undergo hyperpolarization and failed to capacitate and fertilize. The frozen samples hyperpolarized and all of them were capable of fertilizing an egg.

### Membrane Potential May Be Used to Predict Fertility Success

**FIGURE 6:**
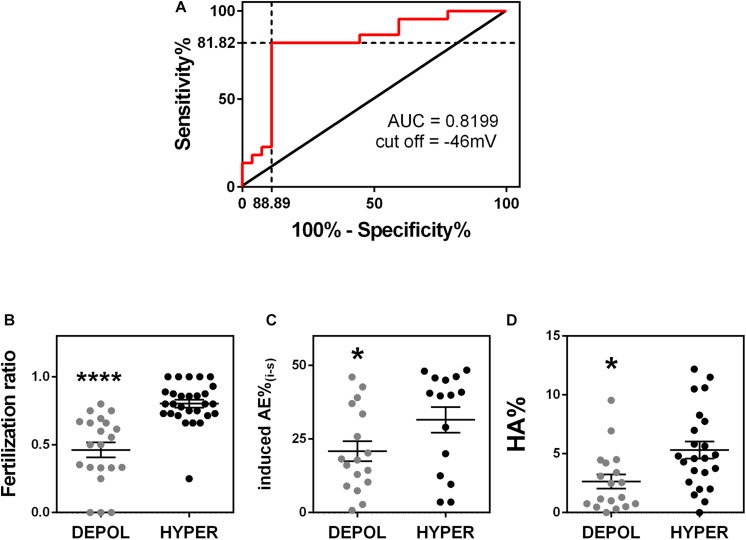
Membrane potential predicts fertility success. **(A)** Receiver operating characteristic curve analysis of model including the sperm membrane potential from 49 normozoospermic infertile patients undergoing conventional *in vitro* fertilization. A fertilization ratio >0.7 was considered positive and <0.7 was considered negative. Area under the curve = 0.8199, *P* = 0.0014. The optimal cut-off value was –45.68 mV (81.82% sensitivity and 88.89% specificity). **(B–D)** Considering the cut off-value of –46 mV, sperm samples were classified as hyperpolarized (HYPER, membrane potential more negative than –46 mV) or depolarized (DEPOL, membrane potential values more positive than –46 mv) and compared to panel **(B)** fertilization ratio (*n* = 49, ^∗∗∗∗^*P* < 0.0001); **(C)** induced acrosomal exocytosis (*n* = 32, ^∗^*P* = 0.049); and **(D)** hyperactivated motility (*n* = 41, ^∗^*P* = 0.031). All *P*-values were calculated by a Kolmogorov-Smirnov test.

Finally, we wanted to determine whether membrane potential values could be used to predict sperm capacitation state and fertilization success. To do this, we first determined a value of membrane potential that allowed us to characterize a sample as either hyperpolarized or depolarized. We used the receiving operating characteristic curve and the maximum Youden index to determine the optimal cut-off value. We considered samples with fertilization ratio >0.7 as the reference for a positive outcome and <0.7 as a negative outcome. The optimal cut-off with 81.82% (59.72 to 84.81%) sensitivity and 88.89% (70.84 to 97.65%) specificity was −46 mV (Figure 6A). We then applied this cut-off value and found that those samples with a sperm membrane potential value more hyperpolarized than −46 mV had a significantly higher IVF fertilization ratio than those with a sperm membrane potential value more depolarized than −46 mV (0.46 ± 0.06, *n* = 21, vs. 0.80 ± 0.03, *n* = 28) (Figure 6B). Furthermore, the percentages of sperm that underwent induced acrosomal exocytosis (Figure 6C) and hyperactivated motility (Figure 6D) were significantly higher in samples with sperm membrane potential values more hyperpolarized than −46 mV. However, ICSI fertilization ratios were not significantly different between sperm samples with membrane potential values more positive and more negative than −46 mV (Supplementary Figure S2). This result is not surprisingly because ICSI bypasses sperm capacitation.

## Discussion

Here, we report a new method that combines flow-cytometry with the voltage sensitive dye DiSC_3_(5) to quantify human sperm membrane potential. This technique could be developed as a new tool to predict sperm fertility capacity. This method has several advantages over other available methods to evaluate the ability of human sperm to capacitate. It is less subjective and time consuming than the CapScore test, which assays the localization pattern of a ganglioside (GM1) within the plasma membrane as a marker of capacitation. This method requires staining, counting, and correctly identifying staining patterns in more than 150 individual sperm (Moody et al., 2017). Patch clamp can be used to measure sperm membrane potential, but the flow cytometry method is simpler and can evaluate membrane potential in higher number of sperm than this technique (Brown et al., 2016). Finally, our flow cytometry method excludes non-viable sperm and requires a smaller sperm sample than does spectrophotometry (0.25 × 10^6^ sperm vs. 4 × 10^6^ sperm).

We argue that quantification of human sperm membrane potential by flow cytometry is a valid method to predict human sperm fertility success for several reasons. First, hyperpolarization is a key event in sperm becoming competent to fertilize an egg in many mammalian species (Zeng et al., 1995; Hernández-González et al., 2007; López-González et al., 2014). In murine and bovine sperm, lack of hyperpolarization is associated with failure to undergo acrosomal exocytosis (Zeng et al., 1995; De La Vega-Beltran et al., 2012). An increase in membrane K^+^ permeability and the subsequent sperm plasma membrane hyperpolarization are essential for sperm capacitation in mice (Chávez et al., 2013). Furthermore, human sperm also hyperpolarize under capacitating conditions (López-González et al., 2014). Brown et al. showed an association between depolarized membrane potential values and poor fertilization rate, as 2 of 19 IVF patients that they analyzed had highly depolarized membrane potential and a low fertilization ratio (Brown et al., 2016).

Second, we validated our method by showing that it produced similar values for wild-type and *Slo3*^–/–^ mouse sperm as have been reported by other methods (Santi et al., 2010). Third, the mean membrane potential value of human sperm from donors capacitated in commercial IVF media (−57.7 ± 4.1 mV, *N* = 22, Supplementary Figure S3) is consistent with the value reported by Patrat et al. (2002) from spectrofluorimetric assays (−57.8 ± 2.20 mV, *n* = 12). In addition, we obtained similar values of human sperm membrane potential by spectrophotometry and flow cytometry. Fourth, the mean value of sperm membrane potential we calculated from patients with fertilization ratio <0.7 (−40.61 ± 2.60 mV) was similar to the value Calzada and Tellez (1997) obtained via a radioactivity assay of sperm from patients with idiopathic infertility (−35.00 ± 1.60 mV). Our value was also similar to the mean membrane potential value of non-capacitated sperm that Linares-Hernández et al. (1998) measured with spectrophotometry (−40.00 ± 6.00 mV). Conversely, we found that membrane potential values of capacitated sperm from patients with successful IVF (−58.03 ± 3.00 mV), from frozen fertile samples (−62.5 ± 4.1 mV), and from fertile donors included in this study (−64.1 ± 7.9 mV *n* = 4, dark gray, Supplementary Figure S3) were similar to the reported values of sperm membrane potential from fertile donors, measured by a radioactivity assay (Calzada −75.0 ± 1.9 mV, *n* = 10).

Fifth, we argue that our method is valid because we found that human sperm, like mouse sperm, hyperpolarized when incubated in capacitating media. However, the extent of this hyperpolarization was smaller in human sperm (mean difference 9.24 mV) than in mouse sperm (mean difference ∼15–30 mV) (Espinosa and Darszon, 1995; Zeng et al., 1995; Muñoz-Garay et al., 2001; Demarco et al., 2003; Hernández-González et al., 2006; Santi et al., 2010; De La Vega-Beltran et al., 2012). This difference in membrane potential between capacitated and non-capacitated human sperm is similar to the 5 mV difference reported by using the patch clamp technique (Brown et al., 2016). It is also noteworthy that human capacitated sperm membrane potential values were more heterogeneous than were mouse capacitated sperm values (coefficients of variation 25.7% vs. 0.29%, respectively), even though all the samples analyzed were normozoospermic (≥32% progressive motility; ≥40% total motility; ≥15 × 10^6^ cells/ml). This variability, along with the small difference in membrane potential values between capacitated and non-capacitated sperm, could explain why changes in membrane potential of human sperm during capacitation are difficult to detect by other methods (Puga Molina et al., 2018a).

Sixth, by using this methodology, we found that membrane hyperpolarization positively correlated with the two endpoints of sperm capacitation: hyperactivated motility and acrosomal exocytosis. Hyperpolarization of the plasma membrane is necessary and sufficient for acrosomal exocytosis of mouse sperm (De La Vega-Beltran et al., 2012), but this is the first report that correlates membrane potential with both hyperactivation and the acrosomal exocytosis and highlights the importance of membrane potential in human sperm capacitation.

Finally, we reported here that hyperpolarization measured by our method correlates with IVF success. Only two previous studies attempted to find an association between membrane potential values and human fertility, but the techniques used to measure sperm membrane potential (patch clamp or radioactivity assays) are extremely challenging and not suitable to clinical implementation (Calzada and Tellez, 1997; Brown et al., 2016). Brown et al. showed that sperm plasma membrane depolarization, measured with the patch clamp technique, was associated with low IVF rates. However, these authors did not report a correlation between the values of membrane potential and fertilization ratio. Additionally, they did not address whether the mean values of sperm membrane potential from successful and unsuccessful IVF significantly differed (Brown et al., 2016). Interestingly our results agree with the results also reported in this issue which were independently obtained by Dr. Krapf’s group showing that IVF rates correlate to human sperm membrane potential values measured by the spectrophotometry technique (Baro Graf et al., 2020).

Until now, the fluorescent probes DiSC3(5) or DiSBAC2(3) have only been used in combination with flow cytometry to report relative changes in sperm membrane potential (López-González et al., 2014; Escoffier et al., 2015; Puga Molina et al., 2017, 2018b; Brukman et al., 2019) and not absolute values. However, we were able to build upon spectrofluorimetric assays in which DiSC_3_(5) fluorescence was conveniently calibrated by using valinomycin and different KCl concentrations, permitting measurement of sperm membrane potential values (Zeng et al., 1995; Demarco et al., 2003; Chávez et al., 2013). Importantly, DiSC_3_(5) has no toxic effects on sperm function at the concentrations used (López-González et al., 2014). When constant concentrations of sperm and dye are used, DiSC_3_ provides reproducible estimates of plasma membrane potential and good signal-to-noise ratio. One potential challenge with using DiSC_3_(5) is that it binds to mitochondria in their normal energized state, and the resulting mitochondrial fluorescence could contribute to the fluorescence signal (Rink et al., 1980). However, we previously measured DiSC_3_(5) fluorescence in the presence and absence of several mitochondrial uncouplers (CCCP, antimycin, and oligomycin) and showed that, in all conditions, the plasma membrane potential of valinomycin-treated mouse sperm responds to the external K^+^ concentration in accordance with the Nernst equation (Chávez et al., 2013). In addition, Baro Graf et al. (2019) showed that the mitochondrial electron transport inhibitor rotenone does not affect DiSC_3_(5) membrane potential measurements in human sperm.

In summary, our data indicate that the absolute value of sperm membrane potential is a good indicator of human sperm fertilizing ability. We found that a membrane potential value of −46 mV could be used to classify the sperm samples as depolarized or hyperpolarized and predict their fertilization success. However, a larger cohort of patients is needed to determine a more accurate cut-off value of membrane potential for use in a clinical setting. Our findings also suggest the possibility of using capacitated sperm membrane potential values as a novel tool to diagnose idiopathic normozoospermic male infertility. In addition, this method could be developed as a fast, simple, clinically feasible assay to personalize fertility treatment plans for normozoospermic infertile men (Oehninger et al., 2014; Palermo et al., 2015). For example, normozoospermic men whose sperm have hyperpolarized values of membrane potential could be guided by clinicians to approaches such as intrauterine insemination or conventional IVF. Conversely, men with sperm with depolarized values of membrane potential might be able to avoid the emotional, physical, and financial costs associated with repeated intrauterine or IVF cycles by being guided to ICSI. Given that few IVF clinics are likely to have a flow cytometer, future work will be aimed at developing a more automated method to measure sperm membrane potential.

## Data Availability Statement

All data generated in this study are included in the article/Supplementary Material.

## Ethics Statement

The studies involving human participants were reviewed and approved by Washington University Institutional Review Board. The patients/participants provided their written informed consent to participate in this study. The animal study was reviewed and approved by Animal Care and Use Committee at Washington University.

## Author Contributions

LP, CS, SG, and PL conceived and planned the experiments. LP and SG performed the experiments, analyzed the data, and obtained consent from patients. LP and CS discussed the results and wrote the manuscript. AB-A performed spectrophotometry assays and helped with mouse experiments. All authors contributed to revising the manuscript and read and approved the submitted version.

## Conflict of Interest

The authors declare that the research was conducted in the absence of any commercial or financial relationships that could be construed as a potential conflict of interest.
