# Supplementary figures and images for: Membrane Potential Determined by Flow Cytometry Predicts Fertilizing Ability of Human Sperm
Source: Front Cell Dev Biol. 2020 Jan 21;7:387. doi: 10.3389/fcell.2019.00387 (PMC6985285; doi:10.3389/fcell.2019.00387)

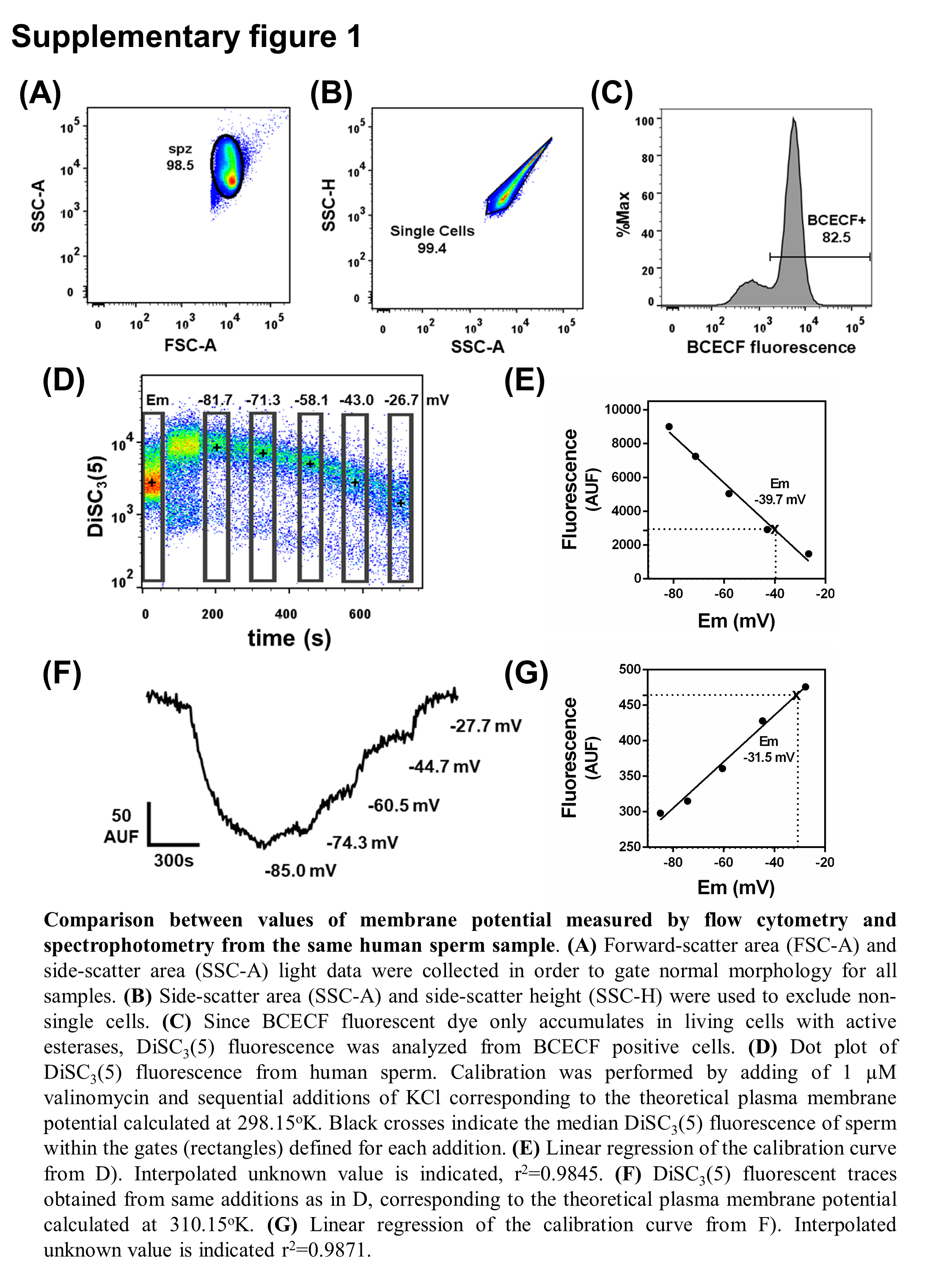

Supplement: Supplementary file 1 [file Image_1.TIF]

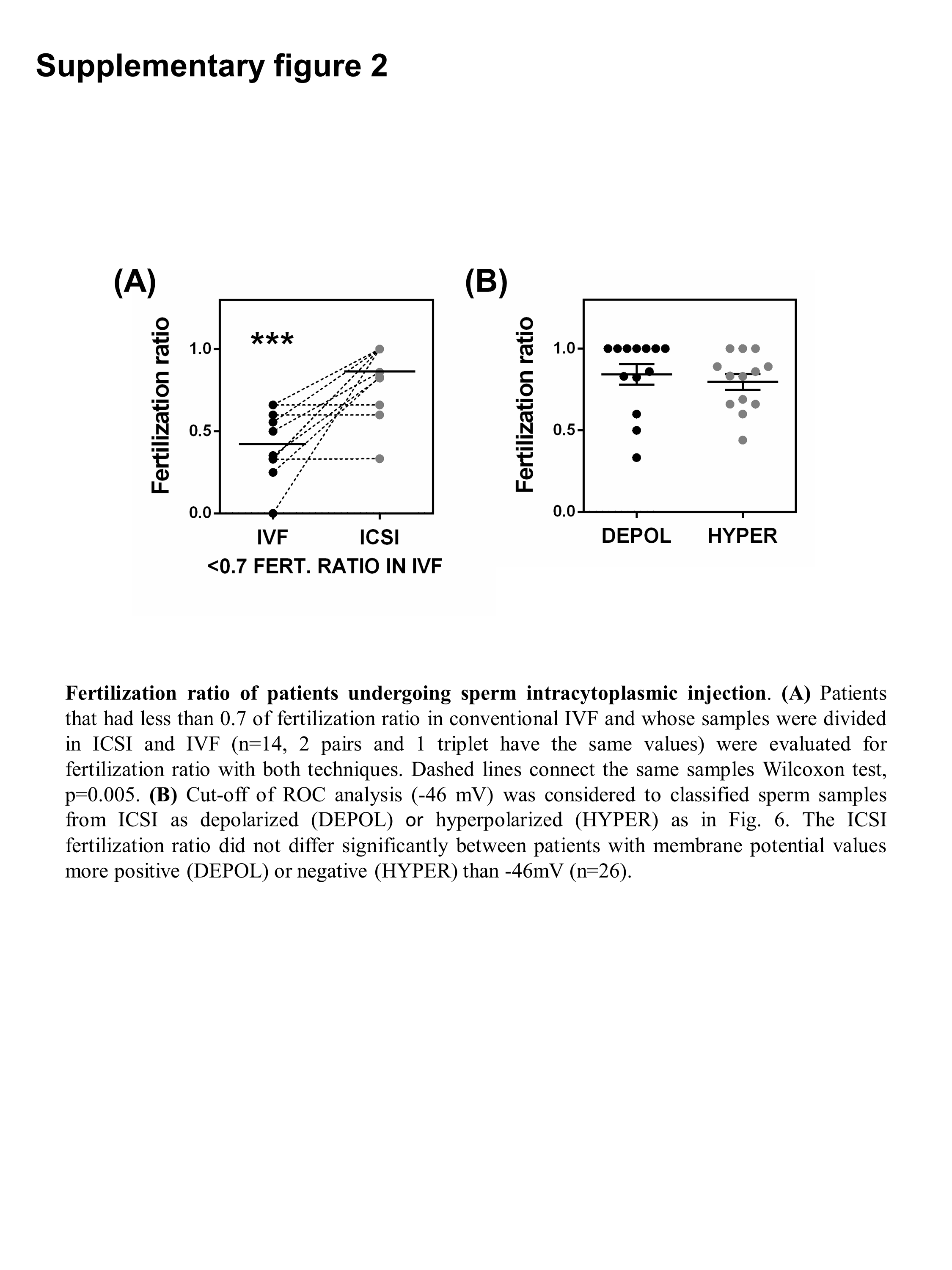

Supplement: Supplementary file 2 [file Image_2.TIF]

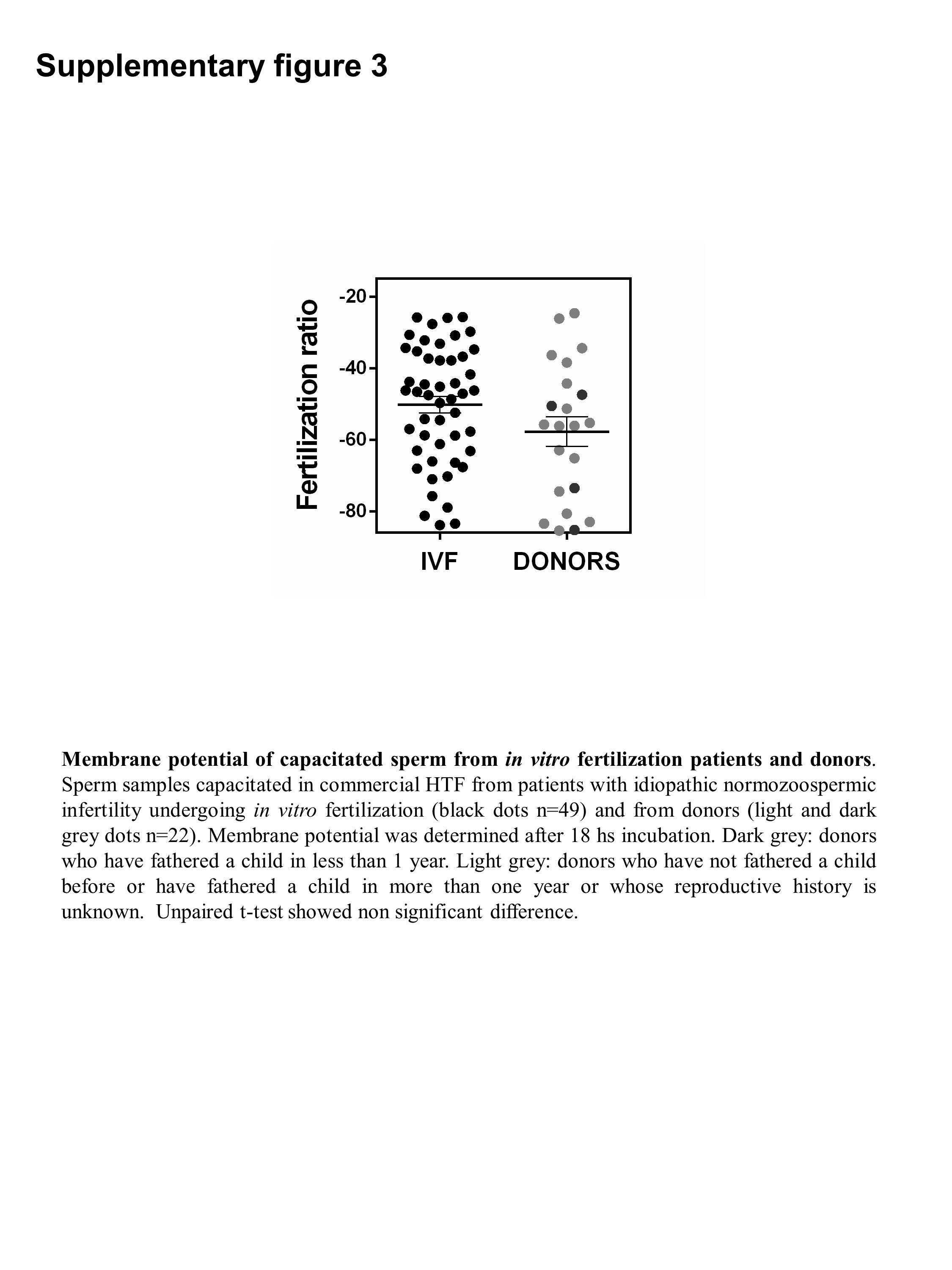

Supplement: Supplementary file 3 [file Image_3.TIF]
